# APOC3 Promotes DGAT2-Dependent Triglyceride Accumulation in Hepatocytes During Early Metabolic Dysfunction

**DOI:** 10.3390/biom16040607

**Published:** 2026-04-20

**Authors:** Thi Nhi Nguyen, Hye-Jeong Kim, Hye Min Shim, Junho Kang, Eun Young Ha, Hochan Cho, Jae-Hyung Park

**Affiliations:** 1 Department of Physiology, School of Medicine, Keimyung University, Daegu 42601, Republic of Korea; nguyenthihhiydh@gmail.com (T.N.N.); abghj0222@gmail.com (H.-J.K.); hmshim1110@gmail.com (H.M.S.); 2 Department of Research, Dongsan Medical Center, Keimyung University, Daegu 42601, Republic of Korea; junho6399@dsmc.or.kr; 3 Department of Internal Medicine, School of Medicine, Keimyung University, Daegu 42601, Republic of Koreaho3632@dsmc.or.kr (H.C.)

**Keywords:** apolipoprotein C-III, DGAT2, triglyceride accumulation, hepatocyte, early metabolic dysfunction, metabolic dysfunction-associated steatotic liver disease

## Abstract

Metabolic dysfunction-associated steatotic liver disease (MASLD) is characterized by hepatic triglyceride accumulation in the setting of obesity and insulin resistance. Although apolipoprotein C-III (APOC3) is a well-established regulator of plasma triglyceride metabolism, its hepatocyte-intrinsic role in intracellular lipid accumulation remains unclear. In this study, we investigated whether APOC3 contributes to hepatocellular triglyceride synthesis during early metabolic dysfunction. In 6-week-old db/db mice, early hepatic lipid accumulation was observed without detectable fibrosis. Transcriptomic profiling identified *APOC3* as an upregulated gene associated with lipid metabolic pathways, and its hepatic upregulation was confirmed at both mRNA and protein levels. Gain- and loss-of-function experiments in HepG2 cells demonstrated that APOC3 overexpression significantly increased intracellular triglyceride content, whereas APOC3 knockdown reduced triglyceride accumulation. Mechanistically, APOC3 selectively regulated diacylglycerol acyltransferase 2 (DGAT2), which catalyzes the final step of triglyceride synthesis, without significantly affecting major lipogenic transcription factors. Furthermore, under de novo lipogenesis-inducing conditions triggered by the liver X receptor agonist T0901317 and insulin, APOC3 markedly amplified DGAT2 expression and triglyceride accumulation. Collectively, these findings suggest a hepatocyte-intrinsic role for APOC3 in promoting triglyceride accumulation through DGAT2-dependent mechanisms. The APOC3–DGAT2 axis may represent a relevant pathway contributing to hepatic lipid accumulation in metabolic liver disease.

## 1. Introduction

Metabolic dysfunction-associated steatotic liver disease (MASLD), formerly termed nonalcoholic fatty liver disease [1], is now the most common chronic liver disorder [2] and is strongly linked to obesity, insulin resistance, and type 2 diabetes [3]. A defining pathological feature of MASLD is the accumulation of triglycerides within hepatocytes, which can precede hepatic inflammation, fibrosis, and progressive liver injury [4,5]. Although hepatocellular triglyceride accumulation is a key initiating event in steatotic liver disease, the molecular basis underlying early lipid deposition in hepatocytes has not been fully clarified [6,7].

Hepatic triglyceride content is governed by the interplay among fatty acid uptake, de novo lipogenesis (DNL), lipid oxidation, and triglyceride export [8,9,10]. Among these processes, triglyceride synthesis is a major determinant of hepatic lipid storage [9,11]. The final step of triglyceride synthesis is catalyzed by diacylglycerol acyltransferase (DGAT) enzymes, which convert diacylglycerol and fatty acyl-CoA into triglycerides [12]. Two DGAT isoforms, DGAT1 and DGAT2, catalyze the same biochemical reaction but appear to play distinct roles in lipid metabolism [12,13]. DGAT2 is abundantly expressed in the liver and is considered an important regulator of hepatic triglyceride synthesis and very-low-density lipoprotein (VLDL) production [12,14]. Experimental studies have shown that inhibition or genetic deletion of *DGAT2* reduces hepatic triglyceride accumulation, supporting its role in hepatic steatosis and its potential as a therapeutic target in metabolic liver disease [15,16].

Apolipoprotein C-III (APOC3) is a small apolipoprotein primarily produced in the liver and intestine and is widely recognized as a key regulator of plasma triglyceride metabolism [17,18]. APOC3 inhibits lipoprotein lipase activity and delays the clearance of triglyceride-rich lipoproteins, thereby promoting elevated circulating triglyceride levels [10,18]. Genetic studies have shown that loss-of-function variants in APOC3 are associated with reduced plasma triglyceride concentrations and decreased cardiovascular risk [19,20], while pharmacological suppression of APOC3 expression has emerged as a promising therapeutic strategy for severe hypertriglyceridemia [18]. In addition to its role in lipoprotein metabolism, APOC3 expression is tightly regulated by metabolic signals [17,18]. Under physiological conditions, insulin suppresses APOC3 transcription and thereby contributes to plasma triglyceride homeostasis [21,22]. During insulin resistance, however, this inhibitory regulation may be weakened, potentially allowing APOC3 expression to increase despite elevated insulin levels [17].

Although APOC3 has been studied extensively in circulating lipoprotein metabolism [17,18], its hepatocyte-intrinsic role in intracellular lipid handling remains poorly understood. In particular, it has not been clearly established whether APOC3 affects hepatic triglyceride synthesis pathways. Because hepatocellular triglyceride accumulation is a key early event in steatotic liver disease [4,5], identifying factors that regulate triglyceride synthesis may provide important insight into hepatic lipid deposition during metabolic dysfunction [6].

In this study, we examined whether APOC3 contributes to hepatic lipid metabolism during early metabolic dysfunction. Using young db/db mice, a model of obesity and insulin resistance, we first assessed hepatic lipid accumulation and performed transcriptomic profiling to identify pathways associated with early steatosis. These analyses revealed increased hepatic APOC3 expression in association with lipid metabolic gene networks. Based on these observations and the insulin-responsive nature of APOC3, we hypothesized that increased APOC3 expression may promote hepatocellular triglyceride accumulation by modulating intracellular lipid synthesis pathways. To test this possibility, we carried out gain- and loss-of-function experiments in hepatocytes to determine whether APOC3 alters triglyceride accumulation and lipid metabolism-related gene expression. Furthermore, because hepatic triglyceride synthesis is strongly influenced by de novo lipogenesis, we examined whether APOC3 modulates hepatocellular lipid accumulation under lipogenic conditions induced by the LXR agonist T0901317 and insulin. Using integrated in vivo and in vitro approaches, we aimed to define the hepatocyte-intrinsic role of APOC3 in hepatic lipid metabolism and to determine whether DGAT2-dependent mechanisms contribute to triglyceride accumulation.

## 2. Materials and Methods

### 2.1. Animal Experiments

Six-week-old male C57BLKS/J-Lepr^db/Lepr^db (db/db) mice were used as a model of early metabolic dysfunction and early steatotic liver disease. Age-matched male C57BLKS/J-m^+/m^+ mice were used as controls. All mice were purchased from Jung-Ang Experimental Animals (Seoul, Republic of Korea) and housed under standard laboratory conditions at 22 ± 2 °C with 55 ± 5% humidity under a 12 h light/dark cycle, with free access to standard chow and water. Following a one-week acclimation period, body weight and fasting blood glucose were recorded before sacrifice. Mice were euthanized at 6 weeks of age, and blood and liver tissues were collected for biochemical, histological, and molecular analyses. A total of 22 mice were used in this study (*n* = 11 per group for biochemical and molecular analyses; *n* = 3 per group for RNA sequencing). All animal procedures were approved by the Institutional Animal Ethics Committee of Keimyung University (approval number: KM-2022-33) and were conducted in accordance with institutional guidelines and the ARRIVE guidelines [23].

### 2.2. Histological and Immunohistochemical Analysis

Liver tissues were fixed in 4% buffered formalin, dehydrated through graded ethanol, and embedded in paraffin. Paraffin blocks were sectioned at a thickness of 5 μm and mounted on glass slides. Hematoxylin and eosin (H&E) staining was performed to evaluate hepatocyte morphology, and Sirius red staining was used to assess collagen deposition. For APOC3 immunohistochemical staining, liver sections were incubated with an anti-APOC3 primary antibody (MBS2028560, MyBioSource, San Diego, CA, USA), followed by appropriate secondary antibodies. Stained sections were visualized using an optical microscope. Positive staining areas were quantified using ImageJ software (version 1.54i; National Institutes of Health, Bethesda, MD, USA).

### 2.3. Measurement of Lipid Profiles

Triglyceride (TG) levels in HepG2 cells, mouse liver tissues, and plasma samples were measured using the CheKine Micro TG Assay Kit (KTB2200, Abbkine, Atlanta, GA, USA) according to the manufacturer’s instructions. Cells or tissues were lysed in extraction buffer and homogenized by sonication. TG concentrations in cellular and tissue samples were normalized to total protein content. Plasma total cholesterol, high-density lipoprotein (HDL) cholesterol, low-density lipoprotein (LDL) cholesterol, and free cholesterol were measured using commercial cholesterol assay kits (ab65390, Abcam, Cambridge, UK) according to the manufacturer’s protocols. Plasma aspartate aminotransferase (AST) and alanine aminotransferase (ALT) levels were measured using the AST Assay Kit (ab263882, Abcam, Cambridge, UK) and the ALT Assay Kit (ab282882, Abcam), respectively, according to the manufacturer’s protocols. Fasting blood glucose levels were measured using the Glucocard Test Strip II (Arkray Inc., Edina, MN, USA). Fasting plasma insulin levels were measured by enzyme-linked immunosorbent assay (ELISA) using the Insulin ELISA Kit (80-INSMS-E01, ALPCO, Salem, NH, USA). The homeostatic model assessment of insulin resistance (HOMA-IR) was calculated using the following formula:

HOMA–IR = [fasting glucose (mg/dL) × fasting insulin (μIU/mL)]/405.

### 2.4. RNA Sequencing and Downstream Bioinformatic Analysis

Total RNA was isolated from mouse liver tissues using TRIzol reagent (Invitrogen, Carlsbad, CA, USA). RNA quality was assessed using a TapeStation 4000 System (Agilent Technologies, Amstelveen, The Netherlands), and RNA quantification was performed using an ND-2000 Spectrophotometer (Thermo Inc., Wilmington, DE, USA). Samples with an RNA Integrity Number equivalent (RINe) ≥7.0 were used for library preparation. Libraries were prepared from total RNA of liver tissues from control mice (*n* = 3) and db/db mice (*n* = 3) using the CORALL RNA-Seq V2 Library Prep Kit (LEXOGEN, Inc., Vienna, Austria) following poly(A) mRNA isolation with the Poly(A) RNA Selection Kit (LEXOGEN, Inc.). Library quality was verified using TapeStation HS D1000 Screen Tape (Agilent Technologies), and library quantification was performed using a StepOne Real-Time PCR System (Life Technologies, Inc., Carlsbad, CA, USA). High-throughput paired-end 100 bp sequencing was performed on a NovaSeq 6000 platform (Illumina, Inc., San Diego, CA, USA), generating more than 4 gigabases of reads per sample. Quality control of raw sequencing data was performed using FastQC v0.12.0, and adapter trimming and low-quality read removal were conducted using Fastp v1.3.2 [24]. Trimmed reads were mapped to the mouse reference genome (GRCm39, NCBI) using STAR [25], and read quantification was processed using Salmon [26]. Read counts were normalized using the TMM + CPM method with edgeR [27]. Differential gene expression analysis and data visualization were performed using ExDEGA (Ebiogen Inc., Seoul, Republic of Korea). Gene enrichment analyses for biological processes and metabolic pathways were performed using STRING and Enrichr databases [28,29]. The top 100 significantly ranked genes were used for gene ontology enrichment, Reactome pathway analysis, and disease–gene network analysis.

### 2.5. Cell Culture

The human hepatoblastoma cell line HepG2 was obtained from the Korean Cell Line Bank (Seoul, Republic of Korea). Cells were cultured in Dulbecco’s Modified Eagle Medium (DMEM; Gibco, New York, NY, USA) supplemented with 10% fetal bovine serum (FBS; Gibco) and 1% penicillin–streptomycin (Thermo Fisher Scientific, New York, NY, USA). Cells were maintained at 37 °C in a humidified incubator containing 5% CO_2_ and seeded into 6-well plates for subsequent experiments.

### 2.6. APOC3 Overexpression

For APOC3 overexpression, HepG2 cells were transfected with an APOC3 expression plasmid (VectorBuilder, Chicago, IL, USA) using Lipofectamine 3000 (Thermo Fisher Scientific) according to the manufacturer’s protocol. DNA–lipid complexes were prepared and incubated for 15 min before addition to the cells. Cells were cultured for 72 h after transfection and were then analyzed under basal conditions or subjected to subsequent DNL induction.

### 2.7. APOC3 Knockdown

APOC3 expression was silenced using APOC3-targeting small interfering RNA (siRNA) obtained from Santa Cruz Biotechnology (sc-41186, Santa Cruz, CA, USA). HepG2 cells were transfected with APOC3-siRNA using Oligofectamine (Thermo Fisher Scientific) according to the manufacturer’s instructions. Negative control siRNA (sc-37007, Santa Cruz Biotechnology) was used as a control. After transfection, cells were incubated with the transfection complex for 24 h and then cultured for an additional 72 h before downstream analyses under basal conditions or after DNL induction.

### 2.8. Induction of De Novo Lipogenesis in HepG2 Cells

To induce de novo lipogenesis (DNL), HepG2 cells were treated with the liver X receptor agonist T0901317 (Sigma-Aldrich, St. Louis, MO, USA) at a final concentration of 1 μM together with insulin (50 nM). Seventy-two hours after APOC3 overexpression or knockdown, cells were exposed to DNL-inducing conditions for an additional 24 h. Vehicle control cells received an equivalent concentration of DMSO. After treatment, cells were harvested for quantitative real-time PCR, Western blot analysis, and intracellular triglyceride measurements.

### 2.9. Western Blot Analysis

Cells and liver tissues were lysed using RIPA lysis buffer or tissue protein extraction reagent (Thermo Fisher Scientific, Rockford, IL, USA) supplemented with protease and phosphatase inhibitors (GenDEPOT, Baker, TX, USA). Protein concentrations were determined using a bicinchoninic acid (BCA) protein assay kit (Thermo Fisher Scientific). Equal amounts of protein were separated on 10% SDS–polyacrylamide gels and transferred to nitrocellulose membranes. Membranes were blocked with 5% skim milk for 1 h at room temperature and incubated with primary antibodies overnight at 4 °C. The following primary antibodies were used: anti-APOC3 (MBS2028560, MyBioSource, San Diego, CA, USA) for mouse samples; anti-APOC3 (sc-293227, Santa Cruz Biotechnology, Santa Cruz, CA, USA) for human samples; anti-vinculin (#13901, Cell Signaling Technology, Danvers, MA, USA); anti-DGAT1 (PA1-16985, Invitrogen, Carlsbad, CA, USA); anti-DGAT2 (PA5-103785, Invitrogen); anti-LIPE (#18381, Cell Signaling Technology); anti-ATGL (#2138, Cell Signaling Technology); anti-CHOP (sc-7351, Santa Cruz Biotechnology); and anti-ATF4 (sc-390063, Santa Cruz Biotechnology). After washing, membranes were incubated with appropriate secondary antibodies, and protein bands were detected using enhanced chemiluminescence. Vinculin was used as the loading control for all Western blot analyses. Band intensities were quantified using ImageJ and normalized to vinculin. Original Western blot images are provided in Supplementary Figures S1–S7.

### 2.10. Quantitative Real-Time PCR

Total RNA was extracted from cells or mouse liver tissues using TRIzol reagent (Invitrogen, Carlsbad, CA, USA). RNA concentration and purity were measured using a spectrophotometer. Complementary DNA (cDNA) was synthesized from total RNA using the High-Capacity cDNA Reverse Transcription Kit (4374967, Applied Biosystems, Foster City, CA, USA). Quantitative real-time PCR was performed using Power SYBR Green PCR Master Mix (Applied Biosystems, Foster City, CA, USA). Relative transcript levels were normalized to *β-actin* (*ACTB*) for human HepG2 cell experiments and to *18S ribosomal RNA* (*18S rRNA*) for mouse liver tissue experiments, and calculated using the 2^−ΔΔCt^ method [30]. Primer sequences used in this study are listed in Supplementary Table S1.

### 2.11. Statistical Analysis

All data are presented as mean ± standard error of the mean (SEM). Statistical analyses were performed using SPSS software (version 25.0; SPSS Inc., Chicago, IL, USA). Comparisons between two groups were performed using two-tailed Student’s *t*-tests. For experiments involving multiple conditions, statistical comparisons were performed between the indicated groups as shown in the figures. A *p*-value < 0.05 was considered statistically significant. All cell experiments were independently repeated at least three times.

## 3. Results

### 3.1. Baseline Metabolic Characteristics of Young db/db Mice

To characterize the metabolic status of the experimental model, baseline metabolic parameters were assessed in 6-week-old db/db mice and age-matched control mice. As shown in Table 1, db/db mice exhibited marked hyperglycemia, with fasting blood glucose levels of 166.91 ± 21.18 mg/dL compared with 82.36 ± 5.56 mg/dL in control mice (*p* < 0.001). Body weight was also significantly higher in db/db mice than in control mice (25.66 ± 0.47 g vs. 17.91 ± 0.25 g, *p* < 0.001). In contrast to these pronounced differences in glucose levels and body weight, plasma lipid profiles were largely similar between the two groups. Levels of plasma total cholesterol, high-density lipoprotein cholesterol, low-density lipoprotein cholesterol, and free cholesterol were not significantly altered in db/db mice relative to control mice. Plasma triglyceride levels also did not differ significantly between db/db mice and control mice, despite a modest numerical increase in the db/db group (66.13 ± 6.79 mg/dL vs. 52.79 ± 5.58 mg/dL, *p* = 0.54). Together, these results show that 6-week-old db/db mice exhibit hyperglycemia and increased body weight in the absence of overt changes in circulating lipid parameters, including plasma triglycerides.

### 3.2. Early Hepatic Lipid Accumulation Without Fibrosis in db/db Mice

**Figure 1:**
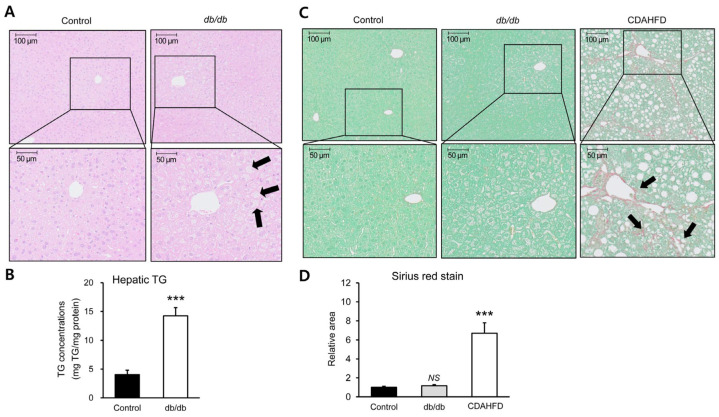
Early hepatic lipid accumulation without fibrosis in db/db mice. (**A**) Hematoxylin and eosin staining of liver tissue from control and db/db mice showing hepatocyte morphology. Ballooned hepatocytes are indicated by black arrows. Scale bars: 50 μm and 100 μm. (**B**) Hepatic triglyceride (TG) content measured using a TG assay kit. (**C**) Representative Sirius red-stained liver sections from control, db/db, and CDAHFD mice for assessment of collagen deposition. Sirius red-positive areas in the CDAHFD group are indicated by black arrows. Scale bars: 50 μm and 100 μm. (**D**) Quantification of Sirius red-positive areas using ImageJ. Data are presented as mean ± SEM. *** *p* < 0.001 vs. control. CDAHFD, choline-deficient high-fat diet; TG, triglyceride; NS, not significant.

To evaluate hepatic alterations associated with early metabolic dysfunction, liver histology and hepatic lipid content were examined in 6-week-old db/db mice. Hematoxylin and eosin staining revealed distinct morphological differences between control and db/db livers. Hepatocytes in control mice exhibited a compact cellular architecture, whereas hepatocytes in db/db mice showed enlarged cell size and cytoplasmic rarefaction, consistent with early lipid accumulation (Figure 1A). Occasional ballooned hepatocytes were also observed in db/db livers, indicating early hepatocellular morphological alterations. Consistent with these histological findings, hepatic triglyceride content was significantly increased in db/db mice compared with control mice (Figure 1B). This increase in hepatic triglyceride levels was observed despite the absence of significant differences in circulating triglyceride levels between the two groups (Table 1). To determine whether these hepatic changes were accompanied by fibrotic remodeling, Sirius red staining was performed to assess collagen deposition. Sirius red-positive areas were comparable between control and db/db mice. In contrast, marked collagen deposition was observed in the choline-deficient high-fat diet (CDAHFD) group, which served as a positive control for hepatic fibrosis (Figure 1C,D). Taken together, these findings demonstrate that 6-week-old db/db mice exhibit hepatic lipid accumulation and early hepatocellular morphological changes in the absence of detectable fibrosis.

### 3.3. Transcriptomic Profiling Reveals Metabolic Reprogramming in the Livers of Young db/db Mice

**Figure 2:**
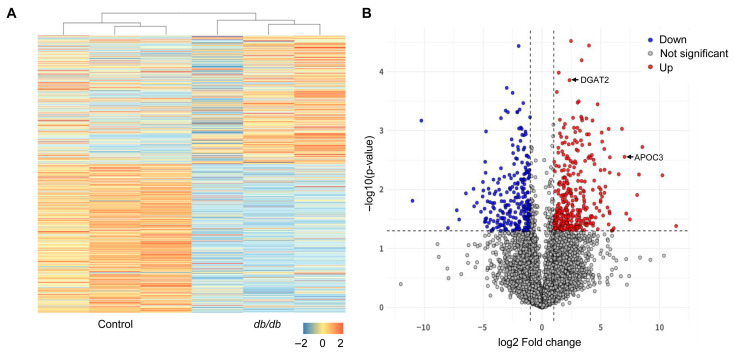
Transcriptomic profiling of liver tissue from young db/db mice. (**A**) Heatmap showing differentially expressed genes in the livers of 6-week-old db/db mice compared with control mice. Gene expression values are presented as row-wise Z-scores. The color scale represents Z-scores ranging from −2 (downregulated) to +2 (upregulated). (**B**) Volcano plot of mRNA expression in db/db versus wild-type control mouse livers constructed using log2 fold-change values and *p*-values. The horizontal black dashed line indicates the threshold for statistical significance (*p* < 0.05). The vertical black dashed lines correspond to 1.5-fold upregulation and downregulation. Red dots represent significantly upregulated genes, and blue dots represent significantly downregulated genes. Key genes discussed in this study, including *APOC3* and *DGAT2*, are individually labeled.

**Figure 3:**
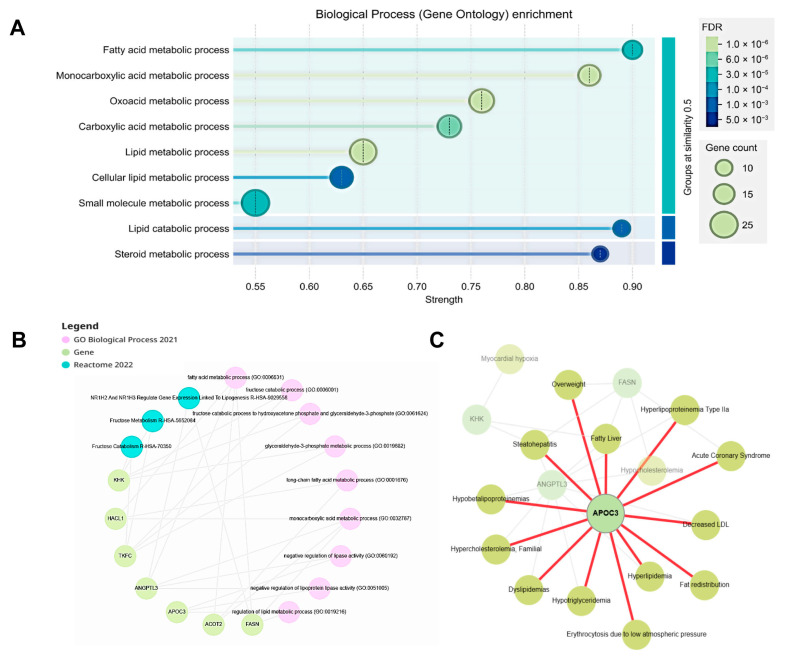
Functional enrichment and disease–gene network analyses of metabolically relevant genes. (**A**) Gene ontology (GO) enrichment analysis of the top 100 differentially expressed genes in the biological process category. (**B**) GO-enriched biological processes and Reactome pathway mapping based on the highest-ranked differentially expressed genes. (**C**) Disease–gene network analysis using DisGeNET, highlighting key metabolic genes and their associated metabolic disorders. ACOT2, acyl-CoA thioesterase 2; ANGPTL3, angiopoietin-like 3; APOC3, apolipoprotein C-III; DisGeNET, disease–gene network analysis; FASN, fatty acid synthase; GO, gene ontology; HACL1, 2-hydroxyacyl-CoA lyase 1; KHK, ketohexokinase; LDL, low-density lipoprotein cholesterol; R-HSA, Reactome of Homo sapiens; TKFC, triokinase mononucleotide cyclase.

To characterize molecular alterations associated with early hepatic lipid accumulation, transcriptomic profiling of liver tissues from 6-week-old db/db mice and control mice were performed using mRNA sequencing. Differential expression analysis identified 257 upregulated and 487 downregulated genes in db/db mouse livers compared with controls, as visualized by heatmap clustering and volcano plot analysis (Figure 2A,B). Gene ontology enrichment analysis revealed significant enrichment of biological processes related to metabolic regulation. In particular, pathways associated with fatty acid metabolism, lipid metabolic processes, and cellular lipid metabolism were prominently represented (Figure 3A), indicating coordinated transcriptional changes in metabolic pathways in db/db mouse livers. To further identify genes contributing to these enriched metabolic pathways, additional enrichment and network analyses were performed. Among the highly ranked genes associated with metabolic regulation were *fatty acid synthase* (*FASN*), *acyl-CoA thioesterase 2* (*ACOT2*), *angiopoietin-like 3* (*ANGPTL3*), *triokinase mononucleotide cyclase* (*TKFC*), *ketohexokinase* (*KHK*), and *apolipoprotein C-III* (*APOC3*) (Figure 3B). These genes are involved in lipid synthesis, fatty acid metabolism, and broader metabolic regulation. Disease–gene network analysis further revealed that several of these genes, including *APOC3*, were connected to metabolic disorder-related disease nodes such as dyslipidemia, hyperlipoproteinemia, fatty liver, and steatohepatitis (Figure 3C). Although this analysis does not establish causality, it highlights *APOC3* as one of several metabolically relevant genes associated with disease-related networks in the context of early hepatic metabolic dysregulation. Taken together, transcriptomic profiling revealed widespread metabolic gene expression changes in the livers of young db/db mice. Within this transcriptional landscape, *APOC3* emerged as a lipid metabolism-associated gene linked to enriched metabolic pathways and disease-related networks.

### 3.4. Altered Expression of Hepatic Apolipoproteins in Early Metabolic Dysregulation

**Figure 4:**
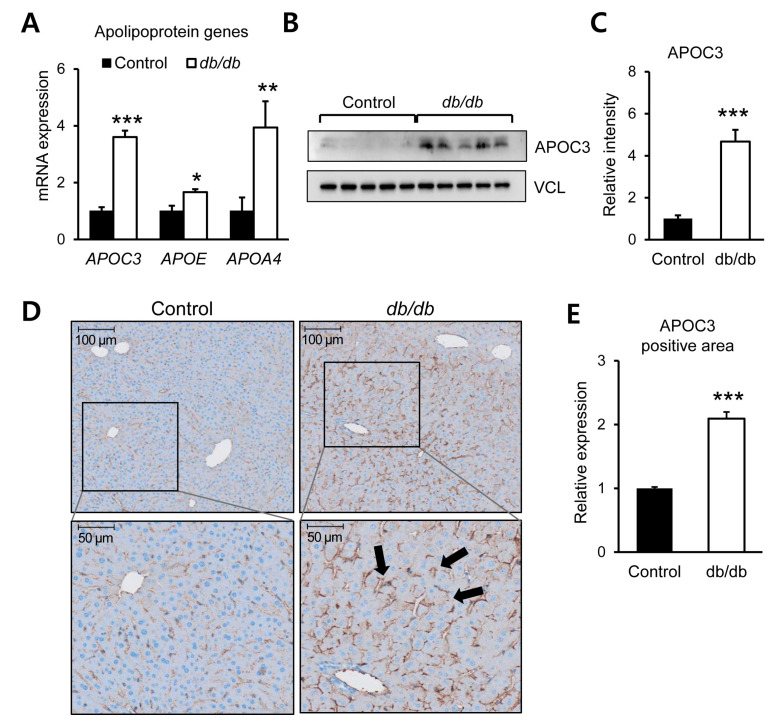
Increased hepatic expression of apolipoproteins in db/db mice. (**A**) Hepatic mRNA expression levels of APOC3, APOE, and APOA4 in 6-week-old db/db mice compared with control mice. (**B**) Representative Western blot analysis of hepatic APOC3 protein expression. (**C**) Densitometric quantification of APOC3 protein levels normalized to vinculin (VCL). (**D**) Representative immunohistochemical staining showing APOC3-positive areas (black arrows) in liver sections from control and db/db mice. Scale bars: 50 μm and 100 μm. (**E**) Quantification of APOC3-positive areas using ImageJ. Data are presented as mean ± SEM (*n* = 6). * *p* < 0.05, ** *p* < 0.01, *** *p* < 0.001 vs. control. APOA4, apolipoprotein A-IV; APOC3, apolipoprotein C-III; APOE, apolipoprotein E; VCL, vinculin.

Based on transcriptomic analyses identifying apolipoprotein-related genes among those associated with enriched metabolic pathways, hepatic expression of selected apolipoproteins was further examined in 6-week-old db/db mice. Quantitative mRNA analysis revealed that hepatic expression levels of *apolipoprotein C-III* (*APOC3*), *apolipoprotein E* (*APOE*), and *apolipoprotein A-IV* (*APOA4*) were significantly increased in db/db mice compared with control mice (Figure 4A). Among these apolipoproteins, APOC3 showed a consistent increase at both transcript and protein levels. Western blot analysis demonstrated significantly elevated hepatic APOC3 protein expression in db/db mice relative to controls (Figure 4B,C and Supplementary Figure S1). Immunohistochemical staining further confirmed increased APOC3 immunoreactivity in liver sections from db/db mice, with a greater proportion of APOC3-positive areas compared with control liver tissue (Figure 4D,E). Taken together, these findings demonstrate increased hepatic expression of multiple apolipoproteins in young db/db mice, with APOC3 showing a particularly robust increase at both mRNA and protein levels.

### 3.5. Altered Lipid Metabolism and Endoplasmic Reticulum Stress Responses in the Livers of db/db Mice

**Figure 5:**
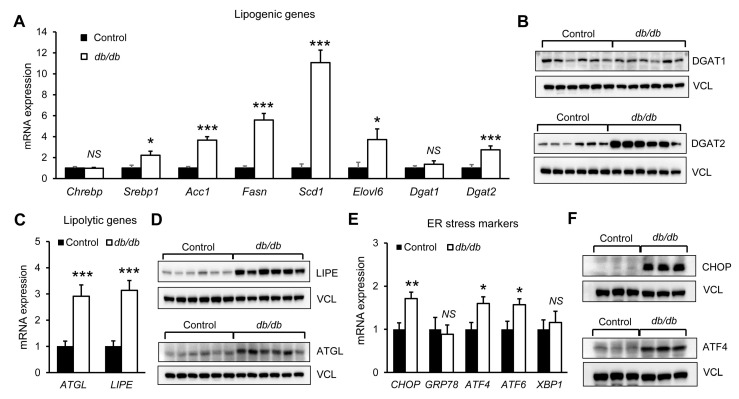
Altered lipid metabolism and endoplasmic reticulum stress responses in db/db mouse liver. Hepatic expression of genes and proteins associated with lipid metabolism and endoplasmic reticulum (ER) stress in 6-week-old db/db mice. Quantitative PCR analysis was used to assess transcripts related to (**A**) lipogenesis, (**C**) lipolysis, and (**E**) ER stress. Data are presented as mean ± SEM (*n* = 6). * *p* < 0.05, ** *p* < 0.01, *** *p* < 0.001 vs. control. Protein abundance of (**B**) lipogenic factors, (**D**) lipolytic factors, and (**F**) ER stress-related proteins was evaluated by Western blotting. ChREBP, carbohydrate-response element–binding protein; SREBP1, sterol regulatory element-binding protein 1; ACC1, acetyl-CoA carboxylase 1; FASN, fatty acid synthase; SCD1, stearoyl-CoA desaturase 1; ELOVL6, elongation-of-very-long-chain fatty acids 6; DGAT1, diacylglycerol acyltransferase 1; DGAT2, diacylglycerol acyltransferase 2; VCL, vinculin; ATGL, adipose triglyceride lipase; LIPE, hormone-sensitive lipase; CHOP, CCAAT-enhancer-binding protein homologous protein; GRP78, glucose-regulated protein 78; ATF4, activating transcription factor 4; ATF6, activating transcription factor 6; XBP1, X-box binding protein 1; NS, not significant.

To further characterize hepatic molecular changes associated with early lipid accumulation, hepatic transcripts related to lipid handling and endoplasmic reticulum (ER) stress were examined in young db/db mice. Quantitative mRNA analysis showed significant upregulation of several lipogenic genes, including *sterol regulatory element-binding protein 1* (*SREBP1*), *acetyl-CoA carboxylase 1* (*ACC1*), *fatty acid synthase* (*FASN*), *stearoyl-CoA desaturase 1* (*SCD1*), and *elongation-of-very-long-chain fatty acids 6* (*ELOVL6*), in db/db mice compared with control mice (Figure 5A). Notably, diacylglycerol acyltransferase 2 (DGAT2) was significantly increased at both the transcript and protein levels, whereas DGAT1 expression remained unchanged relative to control mice (Figure 5A,B and Supplementary Figure S2). In parallel, both mRNA and protein levels of lipolytic genes, including hormone-sensitive lipase (LIPE) and adipose triglyceride lipase (ATGL), were also significantly elevated (Figure 5C,D and Supplementary Figure S3). In addition to these changes in lipid metabolism-related genes, ER stress-associated signals were increased in db/db livers. Hepatic mRNA levels of *CHOP*, *ATF4*, and *ATF6* were significantly higher than those in control mice (Figure 5E). Western blot analysis further showed increased hepatic protein abundance of CHOP and ATF4 (Figure 5F and Supplementary Figure S4). Taken together, these findings indicate coordinated alterations in hepatic lipid metabolism and ER stress signaling in db/db mouse livers.

### 3.6. APOC3 Modulation Differentially Regulates Lipid Accumulation in Hepatocytes Under Basal Conditions

**Figure 6:**
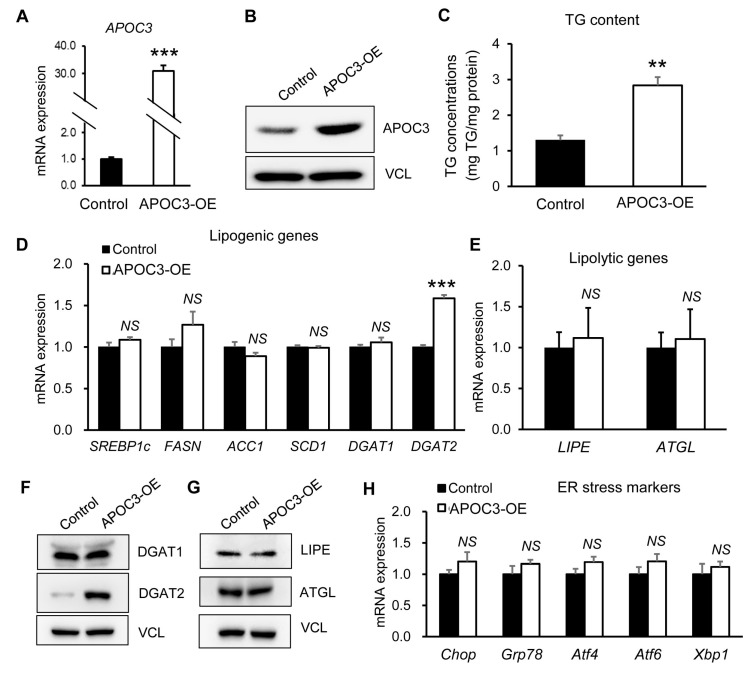
APOC3 overexpression promotes DGAT2-associated triglyceride accumulation in HepG2 cells under basal conditions. (**A**) APOC3 mRNA expression levels in control and APOC3-overexpressing (APOC3-OE) HepG2 cells measured by quantitative PCR. (**B**) APOC3 protein expression in control and APOC3-OE cells assessed by Western blotting. (**C**) Intracellular triglyceride (TG) content in control and APOC3-OE cells measured using a TG assay kit. (**D**) mRNA expression levels of lipogenic genes (SREBP1c, FASN, ACC1, SCD1, DGAT1, and DGAT2). (**E**) mRNA expression levels of lipolytic genes (LIPE and ATGL). (**F**) Protein expression of lipid metabolism-related enzymes (DGAT1, DGAT2, LIPE, and ATGL) determined by Western blotting. (**G**) Densitometric quantification of protein expression normalized to vinculin using ImageJ. (**H**) mRNA expression levels of endoplasmic reticulum (ER) stress markers. Data are presented as mean ± SEM (*n* = 6). ** *p* < 0.01, *** *p* < 0.001 vs. control. APOC3, apolipoprotein C-III; ACC1, acetyl-CoA carboxylase 1; ATGL, adipose triglyceride lipase; DGAT1, diacylglycerol acyltransferase 1; DGAT2, diacylglycerol acyltransferase 2; FASN, fatty acid synthase; LIPE, hormone-sensitive lipase; SCD1, stearoyl-CoA desaturase 1; SREBP1c, sterol regulatory element-binding protein 1c; TG, triglyceride; NS, not significant.

To investigate the hepatocyte-intrinsic role of APOC3, gain- and loss-of-function approaches were performed in HepG2 cells under basal conditions. First, APOC3 overexpression (OE) was verified at the transcript and protein levels (Figure 6A,B and Supplementary Figure S5). APOC3-OE cells exhibited a significant increase in intracellular triglyceride (TG) content compared with control cells (Figure 6C). To explore the molecular basis of this lipid accumulation, the expression of lipid metabolism-related genes was analyzed. APOC3 overexpression did not significantly alter the mRNA levels of canonical lipogenic genes, including *SREBP1c, FASN, ACC1, SCD1*, and *DGAT1*, or lipolytic genes such as *LIPE* and *ATGL* (Figure 6D,E). In contrast, *DGAT2* mRNA expression was significantly increased in APOC3-OE cells compared with control cells (Figure 6D). Consistent with the transcriptional findings, protein analysis showed that DGAT1, LIPE, and ATGL levels were unchanged, whereas DGAT2 protein expression was markedly elevated in APOC3-OE cells (Figure 6F,G and Supplementary Figure S5). In addition, the expression of ER stress-related genes did not differ between control and APOC3-OE cells (Figure 6H).

**Figure 7:**
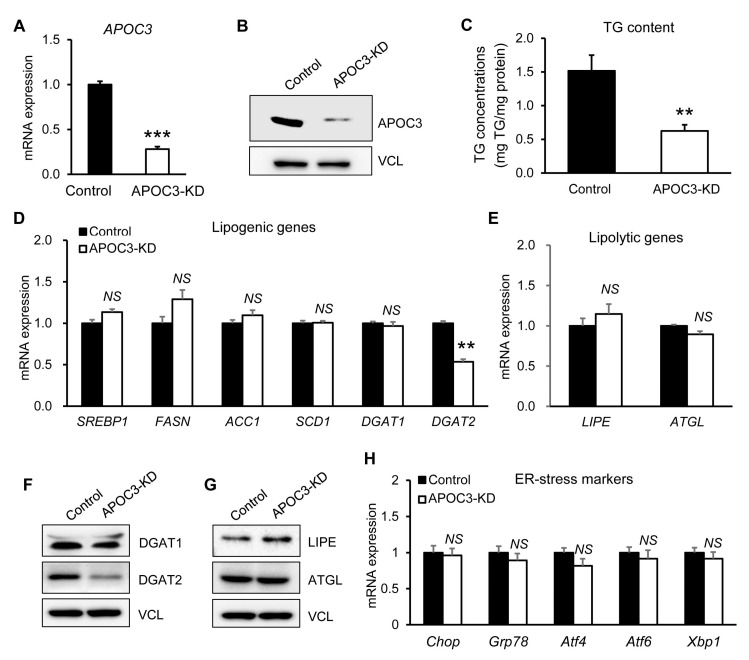
APOC3 knockdown reduces DGAT2 expression and triglyceride accumulation in HepG2 cells under basal conditions. (**A**) APOC3 mRNA expression levels in control and APOC3 knockdown (APOC3-KD) HepG2 cells measured by quantitative PCR. (**B**) APOC3 protein expression in control and APOC3-KD cells assessed by Western blotting. (**C**) Intracellular triglyceride (TG) content in control and APOC3-KD cells measured using a TG assay kit. (**D**) mRNA expression levels of lipogenic genes (SREBP1c, FASN, ACC1, SCD1, DGAT1, and DGAT2). (**E**) mRNA expression levels of lipolytic genes (LIPE and ATGL). (**F**) Protein expression of lipid metabolism-related enzymes (DGAT1, DGAT2, LIPE, and ATGL) determined by Western blotting. (**G**) Densitometric quantification of protein expression normalized to vinculin using ImageJ. (**H**) mRNA expression levels of ER stress markers. Data are presented as mean ± SEM (*n* = 6). ** *p* < 0.01, *** *p* < 0.001 vs. control. APOC3, apolipoprotein C-III; ACC1, acetyl-CoA carboxylase 1; ATGL, adipose triglyceride lipase; DGAT1, diacylglycerol acyltransferase 1; DGAT2, diacylglycerol acyltransferase 2; FASN, fatty acid synthase; LIPE, hormone-sensitive lipase; SCD1, stearoyl-CoA desaturase 1; SREBP1c, sterol regulatory element-binding protein 1c; TG, triglyceride; NS, not significant.

Next, APOC3 knockdown (KD) was induced using APOC3-specific siRNA. Efficient suppression of APOC3 was validated at both transcript and protein levels (Figure 7A,B and Supplementary Figure S6). Under basal conditions, APOC3-KD cells exhibited a significant reduction in intracellular TG content compared with control cells (Figure 7C). Similar to the overexpression results, APOC3 knockdown did not significantly affect the expression of major lipogenic genes (*SREBP1c, FASN, ACC1, SCD1,* and *DGAT1*) or lipolytic genes (*LIPE* and *ATGL*) (Figure 7D,E). However, *DGAT2* transcript levels were significantly reduced in APOC3-KD cells relative to control cells (Figure 7D). Consistent with the mRNA findings, DGAT2 protein expression was also markedly reduced, whereas DGAT1, LIPE, and ATGL protein levels remained unchanged (Figure 7F,G and Supplementary Figure S6). As observed in the overexpression experiments, ER stress-related gene expression was not significantly altered by APOC3 knockdown (Figure 7H).

### 3.7. APOC3 Selectively Amplifies DGAT2-Dependent Triglyceride Accumulation Under De Novo Lipogenesis-Inducing Conditions

**Figure 8:**
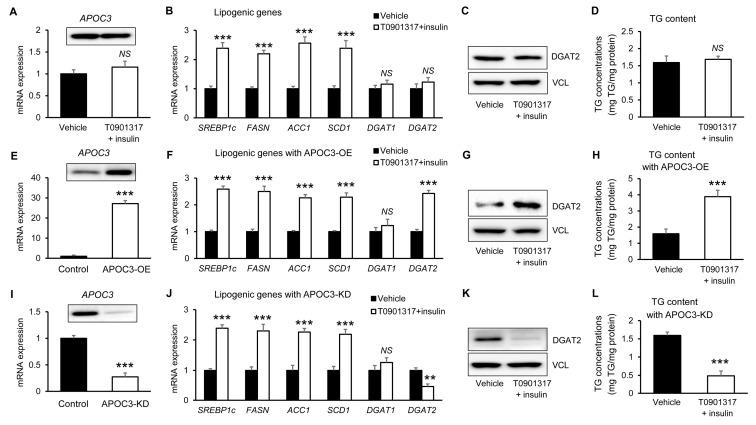
APOC3 selectively enhances DGAT2-dependent triglyceride accumulation under de novo lipogenesis-inducing conditions. De novo lipogenesis (DNL) was induced in HepG2 cells using the LXR agonist T0901317 in combination with insulin to examine whether APOC3 modulates hepatocellular triglyceride accumulation under lipogenic conditions. (**A**–**D**) Effect of DNL induction alone. (**A**) APOC3 transcript and protein abundance in vehicle-treated and DNL-stimulated cells. (**B**) mRNA expression levels of lipogenic genes (SREBP1c, FASN, ACC1, SCD1, DGAT1, and DGAT2). (**C**) DGAT2 protein expression determined by Western blotting. (**D**) Intracellular triglyceride (TG) content. DNL stimulation increased lipogenic gene expression but did not significantly alter APOC3 expression, DGAT2 protein levels, or TG accumulation. (**E**–**H**) Effect of DNL induction in APOC3-overexpressing cells. (**E**) Basal APOC3 expression in control and APOC3-overexpressing (APOC3-OE) HepG2 cells. (**F**) mRNA expression levels of lipogenic genes in APOC3-OE cells treated with vehicle or DNL-inducing conditions. (**G**) DGAT2 protein expression levels in APOC3-OE cells. (**H**) Intracellular TG content. DNL stimulation in APOC3-OE cells markedly increased DGAT2 expression and TG accumulation without altering DGAT1 expression. (**I**–**L**) Effect of DNL induction in APOC3-knockdown cells. (**I**) Basal APOC3 expression in control and APOC3 knockdown (APOC3-KD) HepG2 cells. (**J**) mRNA expression levels of lipogenic genes in APOC3-KD cells treated with vehicle or DNL-inducing conditions. (**K**) DGAT2 protein expression levels in APOC3-KD cells. (**L**) Intracellular TG content. APOC3 knockdown significantly attenuated DGAT2 expression and TG accumulation despite DNL stimulation. Data are presented as mean ± SEM (*n* = 6). ** *p* < 0.01, *** *p* < 0.001 vs. the corresponding control group. ACC1, acetyl-CoA carboxylase 1; APOC3, apolipoprotein C-III; ATGL, adipose triglyceride lipase; DGAT1, diacylglycerol acyltransferase 1; DGAT2, diacylglycerol acyltransferase 2; DNL, de novo lipogenesis; FASN, fatty acid synthase; LIPE, hormone-sensitive lipase; SCD1, stearoyl-CoA desaturase 1; SREBP1c, sterol regulatory element-binding protein 1c; TG, triglyceride; NS, not significant.

To determine whether APOC3 modulates hepatocellular lipid accumulation under lipogenic conditions, de novo lipogenesis (DNL) was induced in HepG2 cells using the LXR agonist T0901317 in combination with insulin. DNL induction alone did not significantly alter *APOC3* mRNA or protein expression compared with vehicle-treated cells (Figure 8A and Supplementary Figure S7). As expected, DNL stimulation significantly increased the expression of lipogenic genes, including *SREBP1c, FASN, ACC1,* and *SCD1*, confirming effective activation of the lipogenic program (Figure 8B). However, *DGAT1* and *DGAT2* mRNA levels remained unchanged, and no significant differences were observed in DGAT2 protein expression (Figure 8C and Supplementary Figure S7) or intracellular TG content (Figure 8D) between DNL-induced and vehicle-treated cells.

Basal APOC3 modulation was first verified under these experimental conditions. APOC3 overexpression and knockdown were verified at both the transcript and protein levels in HepG2 cells (Figure 8E,I and Supplementary Figure S7). Next, the effects of DNL induction were examined in APOC3-overexpressing cells. Under DNL-inducing conditions, *DGAT2* transcript abundance was significantly elevated in APOC3-OE cells, whereas DGAT1 expression remained unchanged (Figure 8F). Lipogenic genes (*SREBP1c, FASN, ACC1*, and *SCD1*) were also significantly upregulated following DNL induction (Figure 8F). Consistent with the transcriptional results, DGAT2 protein levels were prominently increased in APOC3-OE cells under DNL stimulation (Figure 8G and Supplementary Figure S7). Correspondingly, intracellular TG content was significantly elevated in APOC3-OE cells following DNL induction compared with vehicle-treated cells (Figure 8H).

Finally, the impact of DNL induction was evaluated in APOC3-knockdown cells. While DNL stimulation still increased the expression of lipogenic genes (*SREBP1c, FASN, ACC1*, and *SCD1*) (Figure 8J), *DGAT2* mRNA expression was significantly reduced in APOC3-KD cells compared with control cells (Figure 8J). Consistent with this result, DGAT2 protein expression was decreased in APOC3-KD cells (Figure 8K and Supplementary Figure S7). Notably, intracellular TG accumulation was significantly attenuated in APOC3-KD cells despite DNL stimulation (Figure 8L). These results demonstrate that APOC3 enhances hepatocellular triglyceride accumulation primarily through DGAT2-dependent mechanisms under lipogenic conditions, while the core transcriptional program of de novo lipogenesis remains largely unaffected.

## 4. Discussion

**Figure 9:**
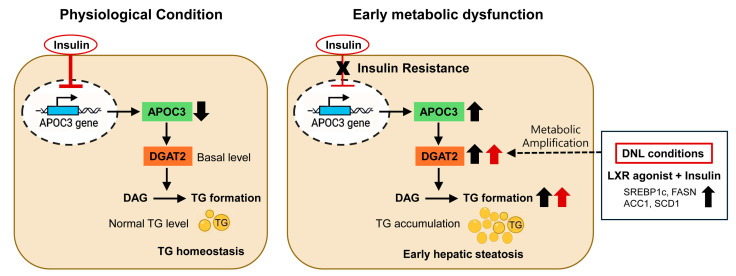
Proposed mechanistic model of APOC3-mediated hepatic triglyceride accumulation during early metabolic dysfunction. Under physiological conditions, insulin suppresses APOC3 transcription in hepatocytes. During the development of insulin resistance, this inhibitory regulation becomes attenuated, leading to increased APOC3 expression. Elevated APOC3 promotes DGAT2 expression, facilitating triglyceride synthesis within hepatocytes. Under lipogenic conditions such as LXR activation and insulin stimulation, this pathway further enhances triglyceride accumulation, contributing to early hepatic steatosis.

The present study suggests that hepatic apolipoprotein C-III (APOC3) expression is increased during early metabolic dysfunction and may be associated with hepatocellular triglyceride accumulation through DGAT2-dependent mechanisms. Using young db/db mice, we identified early hepatic lipid accumulation in the absence of detectable fibrosis, accompanied by transcriptional remodeling of metabolic pathways and increased hepatic APOC3 expression. Functional studies in hepatocytes further showed that modulation of APOC3 expression influenced intracellular triglyceride levels, primarily through selective regulation of DGAT2. In the present study, the term ‘hepatocyte-intrinsic’ refers to the effects arising from altered APOC3 expression within hepatocytes, as distinguished from its well-characterized extracellular role in circulating lipoprotein metabolism, rather than implying a specific subcellular mechanism of action. Notably, this effect became more pronounced under de novo lipogenesis-inducing conditions, suggesting that APOC3 may act as a metabolic amplifier of triglyceride synthesis in hepatocytes exposed to lipogenic stimuli (Figure 9).

Young db/db mice displayed metabolic features consistent with early metabolic dysfunction, including hyperglycemia, increased body weight, and hepatic triglyceride accumulation without overt fibrotic remodeling. This phenotype resembles an early steatotic liver state, in which lipid deposition is already present before inflammatory or fibrotic progression becomes evident [4,5]. Transcriptomic profiling of liver tissues from these mice showed coordinated changes in genes involved in lipid and small-molecule metabolism, indicating that metabolic reprogramming begins before the development of advanced liver pathology. Among the genes identified through transcriptomic analysis, APOC3 emerged as one of the metabolically relevant candidates and showed concordant upregulation at both the transcript and protein levels in db/db mouse livers. Several previous studies have performed transcriptomic profiling of db/db mouse livers at various stages of metabolic dysfunction [31,32]. These studies have consistently demonstrated upregulation of genes involved in lipogenesis, fatty acid metabolism, and inflammatory signaling, along with downregulation of fatty acid oxidation and mitochondrial function-related pathways [31,33]. Our findings are broadly consistent with these prior reports, particularly regarding the coordinated upregulation of lipogenic genes such as *SREBP1, FASN, ACC1*, and *SCD1* in db/db mouse livers. However, the present study differs from previous work in two important aspects. First, we examined the hepatic transcriptome at a very early time point (6 weeks of age), when hepatic lipid accumulation is already present but fibrotic remodeling has not yet developed. Most prior transcriptomic studies of db/db mice were conducted at later stages (8–16 weeks of age), when inflammatory and fibrotic changes are more pronounced [31,32]. Second, our enrichment and network analyses identified APOC3 as a metabolically relevant gene linked to lipid metabolic pathways and disease-related networks, an observation that has not been emphasized in previous db/db liver transcriptomic studies. This may reflect the fact that prior studies focused primarily on canonical lipogenic and inflammatory pathways, whereas our analysis specifically examined apolipoprotein-related gene expression within the broader metabolic transcriptional landscape.

APOC3 is well known as a regulator of plasma triglyceride metabolism and has primarily been studied in the context of lipoprotein regulation [17,18]. Previous studies have shown that APOC3 inhibits lipoprotein lipase activity and impairs clearance of triglyceride-rich lipoproteins, thereby contributing to hypertriglyceridemia [10,18]. Genetic and pharmacological studies have further demonstrated that lowering APOC3 levels reduces circulating triglyceride concentrations and improves lipid profiles [19,20]. By contrast, its contribution to intracellular lipid handling in hepatocytes has not been clearly established. Our findings suggest that APOC3 may exert a hepatocyte-intrinsic effect on triglyceride handling independent of its established role in systemic lipoprotein metabolism.

Mechanistically, our hepatocyte experiments showed that APOC3 selectively regulates DGAT2 expression without significantly altering canonical lipogenic transcription factors such as *SREBP1c* or related downstream lipogenic enzymes. DGAT2 catalyzes the terminal step of triglyceride synthesis by converting diacylglycerol and acyl-CoA into triglycerides and is regarded as a major determinant of hepatic triglyceride synthesis and apoB-containing lipoprotein output [12,14]. In contrast, DGAT1 expression remained unchanged following APOC3 modulation, suggesting that APOC3 influences triglyceride synthesis through a DGAT2-selective pathway rather than broadly activating the de novo lipogenesis transcriptional program. The selective regulation of DGAT2 observed in this study therefore points to a distinct mechanism linking APOC3 expression to hepatic triglyceride synthesis. Importantly, APOC3 knockdown did not significantly alter the expression of ER stress markers (Figure 7H), canonical lipogenic genes, or lipolytic enzymes (Figure 7D–G), indicating that the reduction in triglyceride accumulation was attributable to selective downregulation of DGAT2 rather than to global impairment of cell viability or lipogenic capacity. An important question arising from these findings is how APOC3, a protein classically regarded as a secreted apolipoprotein, may exert intracellular effects on DGAT2 expression. Several possibilities merit consideration. APOC3 is synthesized in the endoplasmic reticulum of hepatocytes, where DGAT2 also resides and catalyzes triglyceride synthesis [12]. Before secretion, newly synthesized APOC3 transits through the ER and Golgi apparatus, during which it may interact with ER-resident proteins or influence ER membrane-associated lipid metabolic processes [34]. It is also recognized that not all newly synthesized apolipoproteins are efficiently secreted; a fraction may be retained intracellularly, particularly under conditions of metabolic stress or impaired VLDL assembly [35]. In this context, intracellular accumulation of APOC3 could potentially modulate lipid metabolic pathways within hepatocytes [36]. Alternatively, APOC3 secreted by hepatocytes may act through autocrine or paracrine signaling via cell surface receptors to influence intracellular gene expression [37]. Precedents for intracellular functions of secreted apolipoproteins have been reported; for example, apolipoprotein E has been shown to affect intracellular lipid metabolism independently of its extracellular lipoprotein-related functions [38]. The present study does not distinguish between these possibilities, and further experiments, including treatment with recombinant APOC3 protein versus intracellular overexpression, subcellular fractionation, and immunofluorescence analyses, will be required to elucidate the precise mechanism by which APOC3 regulates DGAT2 expression in hepatocytes.

Another important aspect of the present findings relates to the interaction between insulin signaling and APOC3 regulation. Under physiological conditions, insulin suppresses APOC3 transcription and thereby contributes to plasma triglyceride homeostasis [21,22]. During the development of insulin resistance, however, this inhibitory effect may become attenuated, allowing APOC3 expression to increase despite elevated insulin levels [17]. In this context, the increased hepatic APOC3 expression observed in young db/db mice may reflect early alterations in insulin signaling associated with metabolic dysfunction. Our hepatocyte data further suggest that APOC3 upregulation may promote triglyceride accumulation through DGAT2-dependent mechanisms. Because DGAT2 catalyzes the terminal step of triglyceride synthesis, its activation can facilitate hepatic lipid storage even in the absence of broad activation of upstream lipogenic pathways [12,16].

Importantly, the effect of APOC3 on triglyceride accumulation became more pronounced under conditions that promote de novo lipogenesis. In our experimental model, stimulation with the LXR agonist T0901317 and insulin increased the expression of lipogenic genes but did not significantly elevate intracellular triglyceride accumulation on its own. The concentrations of T0901317 (1 μM) and insulin (50 nM) used in the present study represent pharmacological conditions commonly employed to activate the LXR–SREBP1c lipogenic pathway in hepatocyte culture systems [9,39]. Although these concentrations exceed physiological levels, they are intended to recapitulate the transcriptional environment of insulin-resistant states, in which LXR activity is enhanced and hepatic lipogenic gene expression is upregulated despite hyperinsulinemia [4,9]. Notably, the lipogenic gene expression profile observed under these in vitro conditions—including upregulation of *SREBP1c, FASN, ACC1*, and *SCD1*—was consistent with the hepatic transcriptional changes observed in 6-week-old db/db mice (Figure 5A), supporting the relevance of this model to the in vivo findings. However, when APOC3 expression was increased under these lipogenic conditions, DGAT2 expression and triglyceride accumulation were markedly enhanced. Conversely, suppression of APOC3 attenuated these effects despite continued activation of lipogenic transcriptional pathways. These observations suggest that APOC3 may function as a metabolic amplifier that facilitates triglyceride storage when lipogenic substrates or signals are abundant. In a physiological context, such a mechanism may help explain why hepatic steatosis can accelerate once insulin resistance and lipogenic signaling become established [4,5].

The present findings may also have translational relevance in the setting of emerging therapeutic strategies targeting lipid metabolism. Pharmacological inhibition of APOC3 has recently attracted attention as a treatment approach for severe hypertriglyceridemia [18]. Antisense oligonucleotide-based therapies targeting APOC3, including volanesorsen and olezarsen, have demonstrated substantial triglyceride-lowering effects in clinical studies [40,41]. Although these agents were primarily developed to modulate circulating lipoprotein metabolism, our findings raise the possibility that APOC3 suppression may also affect hepatocellular triglyceride handling. Likewise, DGAT2 has emerged as a therapeutic target in metabolic liver disease [15,16]. Several DGAT2 inhibitors, including ervogastat (PF-06865571), are currently being evaluated in clinical trials for metabolic dysfunction-associated steatotic liver disease [42]. In both preclinical models and early clinical studies, DGAT2 inhibition has been associated with lower hepatic triglyceride levels and attenuation of steatotic changes [15,42]. In this context, identification of a potential APOC3–DGAT2 regulatory axis in hepatocytes suggests that modulation of this pathway may represent a biologically relevant mechanism influencing hepatic lipid accumulation. From a therapeutic perspective, our findings suggest that APOC3 inhibition may confer benefits beyond the reduction of circulating triglycerides by additionally attenuating hepatocellular triglyceride synthesis through DGAT2-dependent mechanisms. Given that DGAT2 inhibitors are also being developed for the treatment of MASLD [42], the convergence of APOC3 and DGAT2 inhibition on a shared downstream pathway raises the possibility that combination strategies targeting both molecules could offer synergistic effects in reducing hepatic steatosis. Future preclinical studies evaluating the combined effects of APOC3 suppression and DGAT2 inhibition on hepatic lipid accumulation would help clarify the therapeutic potential of this approach.

Several limitations should be acknowledged. First, although our findings indicate that APOC3 regulates DGAT2 expression and triglyceride accumulation in hepatocytes, the upstream signaling pathways linking APOC3 to DGAT2 regulation remain to be defined. Moreover, because the in vivo data were obtained at a single time point (6 weeks of age), it remains unclear whether APOC3 upregulation precedes or is concurrent with hepatic triglyceride accumulation. Time-course experiments and in vivo intervention studies, such as hepatocyte-specific APOC3 knockdown in db/db mice, will be required to establish the temporal and causal relationship between APOC3 upregulation and early hepatic steatosis. In particular, hepatocyte-specific APOC3 knockdown or overexpression in db/db mice using viral vector-mediated gene delivery would provide direct in vivo evidence for the causal role of the APOC3–DGAT2 axis in hepatic triglyceride accumulation. Second, the present study primarily utilized HepG2 cells to investigate hepatocyte-intrinsic mechanisms, and additional studies using primary hepatocytes or in vivo genetic models will be required to further validate these observations. Finally, while the present data support a role for APOC3 in early metabolic dysfunction, further work will be needed to clarify whether this pathway also participates in the transition from early steatosis to more advanced forms of MASLD.

## 5. Conclusions

In summary, this study provides evidence for a hepatocyte-intrinsic role of APOC3 in promoting triglyceride accumulation through DGAT2-dependent mechanisms. Increased APOC3 expression during early metabolic dysfunction may facilitate hepatic triglyceride synthesis, particularly under lipogenic conditions. Together, these results support a mechanistic link between APOC3 upregulation and altered hepatic lipid handling and suggest that the APOC3–DGAT2 axis may represent a potential target for therapeutic intervention in metabolic liver disease.

## Figures and Tables

**Table 1:** Baseline characteristics of 6-week-old db/db mice and control mice.

Parameters	Control	db/db	*p*-Value
Body weight (g)	17.91 ± 0.25	25.66 ± 0.47	*p* < 0.001
Fasting glucose (mg/dL)	82.36 ± 5.56	166.91 ± 21.18	*p* < 0.001
Fasting insulin (µIU/mL)	5.41 ± 0.16	68.37 ± 0.94	*p* < 0.001
HOMA-IR	1.16 ± 0.03	28.25 ± 0.39	*p* < 0.001
Plasma total cholesterol (µg/µL)	2.18 ± 0.05	2.13 ± 0.05	*p* = 0.54
Plasma HDL cholesterol (µg/µL)	0.54 ± 0.02	0.51 ± 0.03	*p* = 0.43
Plasma LDL cholesterol (µg/µL)	2.07 ± 0.13	2.09 ± 0.08	*p* = 0.92
Plasma free cholesterol (µg/µL)	0.45 ± 0.02	0.47 ± 0.02	*p* = 0.56
Plasma TG (mg/dL)	52.79 ± 5.58	66.13 ± 6.79	*p* = 0.54
AST (U/L)	86.32 ± 9.41	99.53 ± 11.27	*p* = 0.13
ALT (U/L)	45.21 ± 9.38	58.37 ± 10.64	*p* = 0.17

Baseline characteristics were determined in control and db/db mice at 6 weeks of age. The values represent the mean ± standard error of mean, *n* = 11. HOMA-IR, homeostatic model assessment of insulin resistance; HDL: high-density lipoprotein; LDL: low-density lipoprotein; TG: triglyceride; AST, aspartate aminotransferase; ALT, alanine aminotransferase.

## Data Availability

The original contributions presented in the study are included in the article/Supplementary Material. Further inquiries can be directed to the corresponding author.

## Supplementary Materials

The following supporting information can be downloaded at: https://www.mdpi.com/article/10.3390/biom16040607/s1, Table S1: Primers for quantitative polymerase chain reaction. Figures S1–S7: Original Western blot images.

## Abbreviations

The following abbreviations are used in this manuscript:

APOC3	Apolipoprotein C-III
DGAT2	Diacylglycerol acyltransferase 2
DNL	De novo lipogenesis
MASLD	Metabolic dysfunction-associated steatotic liver disease
KD	Knockdown
OE	Overexpression
TG	Triglyceride
