# Supplementary material for: APOC3 Promotes DGAT2-Dependent Triglyceride Accumulation in Hepatocytes During Early Metabolic Dysfunction
Source: Biomolecules. 2026 Apr 20;16(4):607. doi: 10.3390/biom16040607 (PMC13113833; doi:10.3390/biom16040607)
Supplement: Supplementary file 1 [file biomolecules-16-00607-s001.zip › Supplementary_table S1.pdf]

Supplementary Material

Supplementary Tables S1. Primers for quantitative polymerase chain reaction

Genes	Sequences of primers
Human *β-actin*	Forward: 5′ - AATCTGGCACCACACCTTCTAC - 3'<br>Reverse: 5′ - ATAGCACAGCCTGGATAGCAAC - 3'
Human *ACC1*	Forward: 5′ - CATGCGGTCTATCCGTAGGTG - 3'<br>Reverse: 5′ - GTGTGACCATGACAACGAATCT - 3'
Human *APOC3*	Forward: 5′ - CCGCCAAGGATGCACTGAG - 3'<br>Reverse: 5′ - CTCCAGTAGTCTTTCAGGGAAC - 3'
Human *ATGL*	Forward: 5′ - ATGGTGGCATTTCAGACAACC - 3'<br>Reverse: 5′ - CGGACAGATGTCACTCTCGC - 3'
Human *ChREBP*	Forward: 5′ - AGAACCGGCGTATCACACAC - 3'<br>Reverse: 5′ - GTGCTCACGAGCCCATGAA - 3'
Human *CYP1A2*	Forward: 5′ - ATGCTCAGCCTCGTGAAGAAC - 3'<br>Reverse: 5′ - GTTAGGCAGGTAGCGAAGGAT - 3'
Human *CYP2E1*	Forward: 5′ - GGGAAACAGGGCAATGAGAG - 3'<br>Reverse: 5′ - GGGAAACAGGGCAATGAGAG - 3'
Human *DGAT1*	Forward: 5′ - GGTCCCCAATCACCTCATCTG - 3'<br>Reverse: 5′ - TGCACAGGGATGTTCCAGTTC - 3'
Human *DGAT2*	Forward: 5′ - AGCAGGTGATCTTCGAGGAG - 3'<br>Reverse: 5′ - CATGGGGCGAAACCAATGTA - 3'
Human *ELOVL6*	Forward: 5′ - AGCAGTCAGTTTGTGACCAGG - 3'<br>Reverse: 5′ - ATCTCCTAGTTCGGGTGCTTT - 3'
Human *FASN*	Forward: 5′ - AAGGACCTGTCTAGGTTTGAT - 3'<br>Reverse: 5′ - TGGCTTCATAGGTGACTTCCA - 3'
Human *LIPE*	Forward: 5′ - TCAGTGTCTAGGTCAGACTGG - 3'<br>Reverse: 5′ - AGGCTTCTGTTGGGTATTGGA - 3'
Human *SCD1*	Forward: 5′ - TTCCTACCTGCAAGTTCTACAC - 3'<br>Reverse: 5′ - CCGAGCTTTGTAAGAGCGGT - 3'
Human *SREBP1*	Forward: 5′ - GCCCCTGTAACGACCACTG - 3'<br>Reverse: 5′ - CAGCGAGTCTGCCTTGATG - 3'

**Genes**	**Sequences of primers**
Mouse *18s rRNA*	Forward: 5′ - GTAACCCGTTGAACCCCATT - 3'<br>Reverse: 5′ - CCATCCAATCGGTAGTAGCG - 3'
Mouse *ACC1*	Forward: 5′ - GATGAACCATCTCCGTTGGC - 3'<br>Reverse: 5′ - GACCCAATTATGAATCGGGAGT - 3'
Mouse *APOA4*	Forward: 5′ - CCAATGTGGTGTGGGATTACT - 3'<br>Reverse: 5′ - AGTGACATCCGTCTTCTGAAAC - 3'
Mouse *APOC3*	Forward: 5′- AGGCTACTGGAGCAAGTTTACT - 3'<br>Reverse: 5′- ATAGCTGGAGTTGGTTGGTCC - 3'
Mouse *APOE*	Forward: 5′ - GACCCAGCAAATACGCCTG - 3'<br>Reverse: 5′ - CATGTCTTCCACTATTGGCTCG - 3'
Mouse *ATF4*	Forward: 5′ - GCAAGGAGGATGCCTTTTC - 3'<br>Reverse: 5′ - GTTTCCAGGTCATCCATTCG - 3'
Mouse *ATF6*	Forward: 5′ - AGCGCCCAAGACTCAAACC - 3'<br>Reverse: 5′ - CTGTATGCTGATAATCGACTGC - 3'
Mouse *ATGL*	Forward: 5′ - ACCACCCTTTCCAACATGCTA - 3'<br>Reverse: 5′ - GGCAGAGTATAGGGCACCA - 3'
Mouse *CHOP*	Forward: 5′ - CTGGAAGCCTGGTATGAGGAT - 3'<br>Reverse: 5′ - CAGGGTCAAGAGTAGTGAAGGT - 3'
Mouse *ChREBP*	Forward: 5′- AGATGGAGAACCGACGTATCA - 3'<br>Reverse: 5′ - ACTGAGCGTGCTGACAAGTC - 3'
Mouse *DGAT1*	Forward: 5′ - GTGCCATCGTCTGCAAGATTC - 3'<br>Reverse: 5′ - GCATCACCACACACCAATTCAG - 3'
Mouse *DGAT2*	Forward: 5′ - TTCCTGGCATAAGGCCCTATT - 3'<br>Reverse: 5′ - CCTCCAGACATCAGGTACTCG - 3'
Mouse *ELOVL6*	Forward: 5′ - TGAACAAGCGAGCCAAGTTTG - 3'<br>Reverse: 5′ - GAGCACCGAATATACTGAAGAC - 3'
Mouse *FASN*	Forward: 5′ - GGAGGTGGTGATAGCCGGTAT - 3'<br>Reverse: 5′ - TGGGTAATCCATAGAGCCCAG - 3'
Mouse *GRP78*	Forward: 5′ - CATCACGCCGTC CTATGTCG - 3'<br>Reverse: 5′ - CGTCAAAGACCGTGTTCTCG - 3'

**Genes**	**Sequences of primers**
Mouse *LIPE*	Forward: 5′ - CCAGCCTGAGGGCTTACTG - 3'<br>Reverse: 5′ - CTCCATTGACTGTGACATCTCG - 3'
Mouse *SCD1*	Forward: 5′ - TTCTTGCGATACACTCTGGTG - 3'<br>Reverse: 5′ - CGGGATTGAATGTTCTTGTCG - 3'
Mouse *SREBP1*	Forward: 5′ - TGACCCGGCTATTCCGTGA - 3'<br>Reverse: 5′ - CTGGGCTGAGCAATACAGTTC - 3'
Mouse *XBP1*	Forward: 5′ - AGCAGCAAGTGGTGGATTTG - 3'<br>Forward: 5′ - GAGTTTTCTCCCGTAAAAGCT - 3'

18s rRNA: 18S ribosomal RNA; *ACC1*: acetyl-CoA carboxylase 1; *APOA4*: apolipoprotein A-IV; *APOC3*: apolipoprotein C-III; *APOE*: apolipoprotein E; *ATF4*: activating transcription Factor 4; *ATF6*: activating transcription factor 4; *ATGL*: adipose triglyceride lipase; *CHOP*: CCAAT-enhancer-binding protein homologous protein; *ChREBP*: carbohydrate-response element binding protein; *CYP1A2*: cytochrome P450 family 1 subfamily A member 2; *CYP2E1*: cytochrome P450 family 2 subfamily E member 1 *DGAT1*: diacylglycerol acyltransferase 1; *DGAT2*: diacylglycerol acyltransferase 2; *ELOVL6*: elongation-of-very-long-chain-fatty acids 6; *ER*: endoplasmic reticulum; *FASN*: fatty acid synthase; *GRP78*: glucose-regulated protein 78; *LIPE*: hormone-sensitive lipase; *SCD1*: stearoyl-coA desaturase 1; *SREBP1*: sterol regulatory element binding protein 1; *XBP1*: x-box binding protein 1.
