# Supplementary material for: APOC3 Promotes DGAT2-Dependent Triglyceride Accumulation in Hepatocytes During Early Metabolic Dysfunction
Source: Biomolecules. 2026 Apr 20;16(4):607. doi: 10.3390/biom16040607 (PMC13113833; doi:10.3390/biom16040607)
Supplement: Supplementary file 1 [file biomolecules-16-00607-s001.zip › Supplementary_Figures.pdf]

Fig 4B

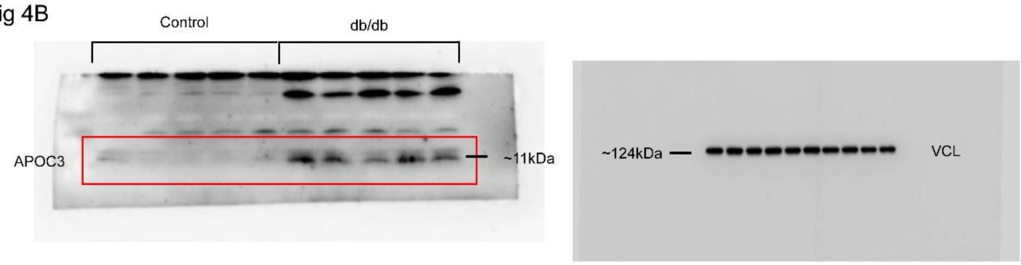

Supplementary Figure S1. Original Western blot images for Figure 4B. Full-length blots showing hepatic APOC3 protein expression in control and db/db mice. Vinculin (VCL) was used as a loading control.

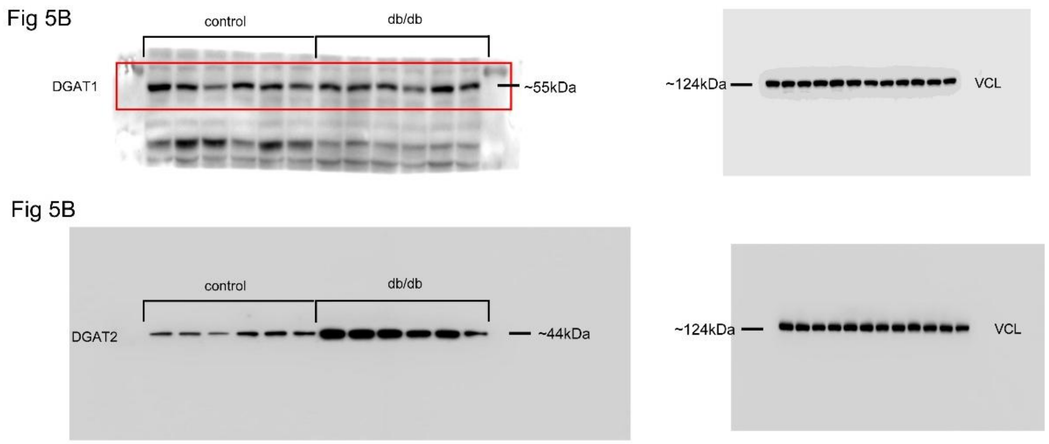

Supplementary Figure S2. Original Western blot images for Figure 5B. Full-length blots showing hepatic protein expression of DGAT1 and DGAT2 in control and db/db mice. Vinculin (VCL) was used as a loading control.

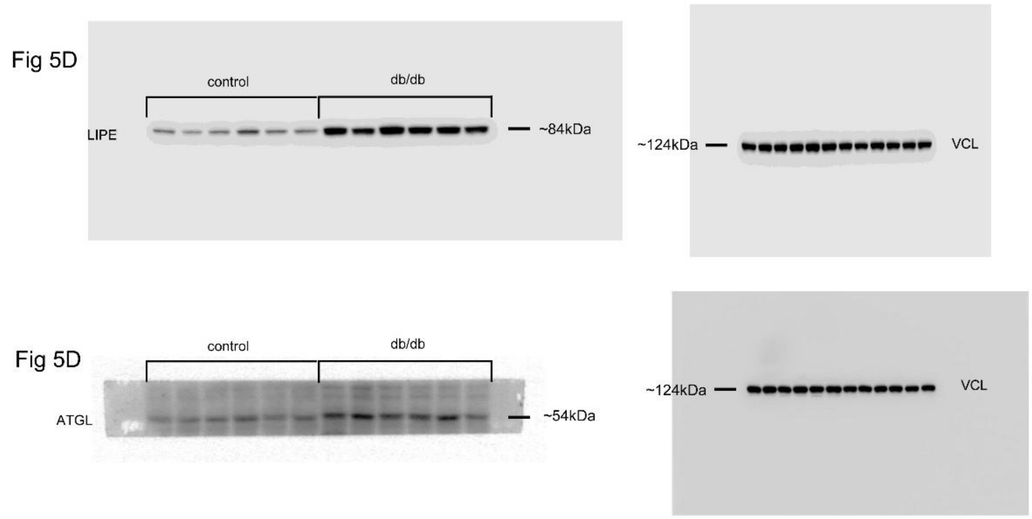

Supplementary Figure S3. Original Western blot images for Figure 5D. Full-length blots showing hepatic protein expression of LIPE and ATGL in control and db/db mice. Vinculin (VCL) was used as a loading control.

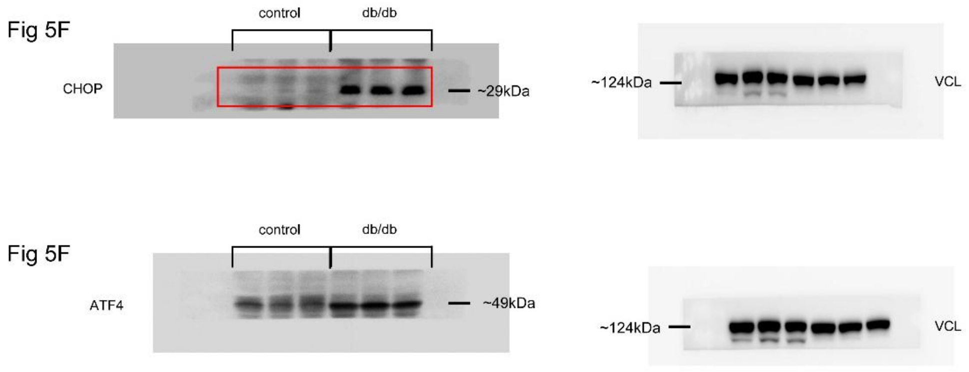

Supplementary Figure S4. Original Western blot images for Figure 5F. Full-length blots showing hepatic protein expression of CHOP and ATF4 in control and db/db mice. Vinculin (VCL) was used as a loading control.

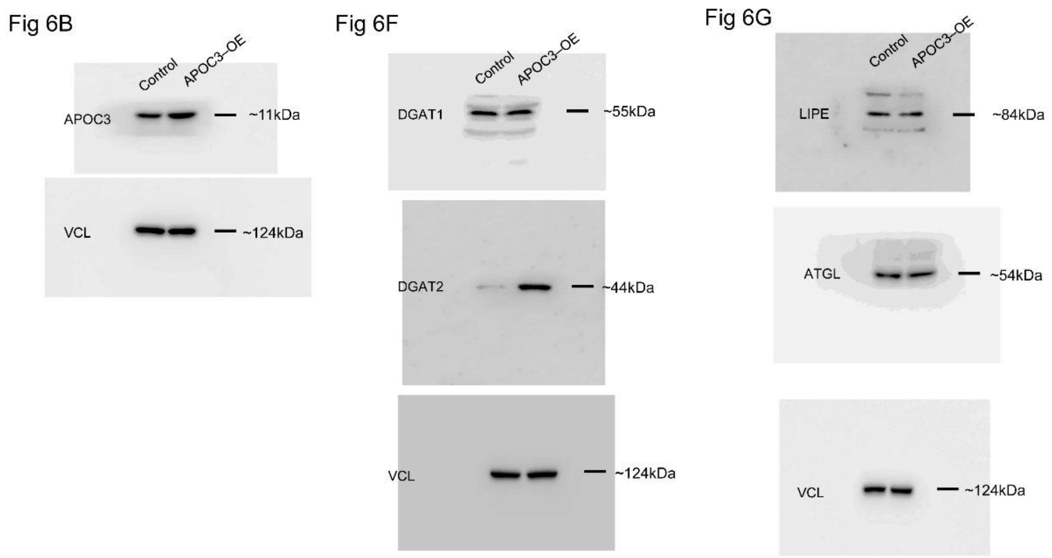

Supplementary Figure S5. Original Western blot images for Figure 6B, F, and G. Full-length blots showing protein expression of APOC3, DGAT1, DGAT2, LIPE, and ATGL in control and APOC3-overexpressing (APOC3-OE) HepG2 cells under basal conditions. Vinculin (VCL) was used as a loading control.

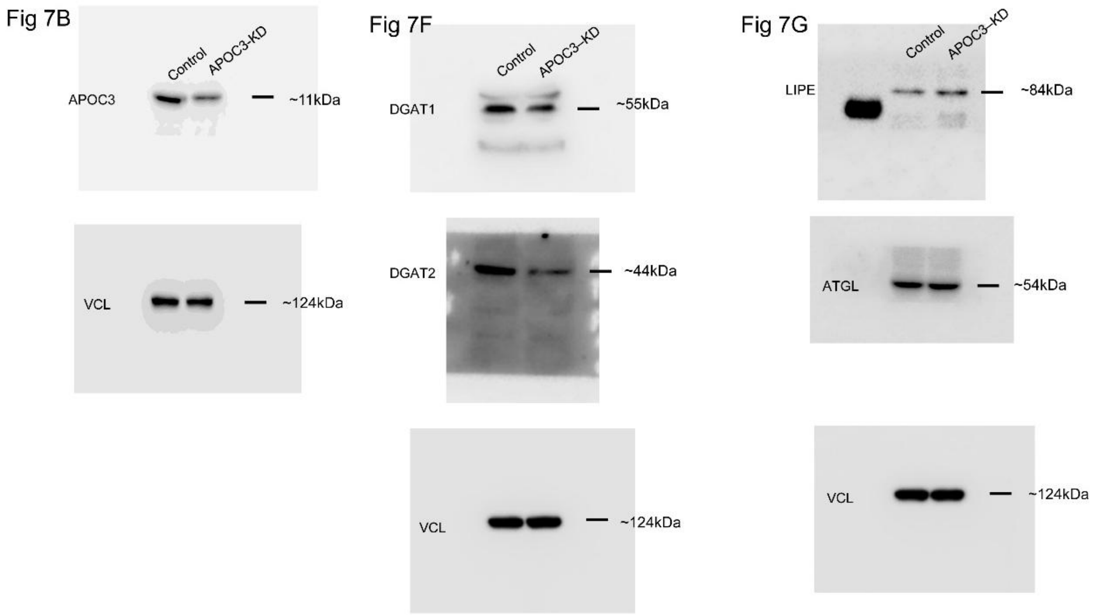

Supplementary Figure S6. Original Western blot images for Figure 7B, F, and G. Full-length blots showing protein expression of APOC3, DGAT1, DGAT2, LIPE, and ATGL in control and APOC3 knockdown (APOC3-KD) HepG2 cells under basal conditions. Vinculin (VCL) was used as a loading control.

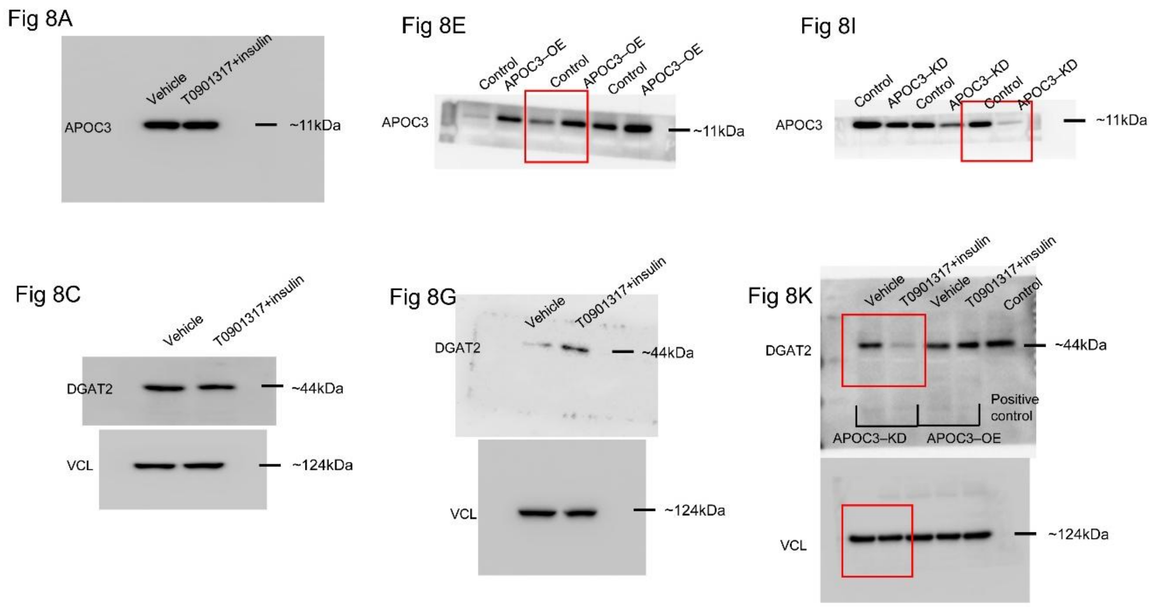

Supplementary Figure S7. Original Western blot images for Figure 8A, C, E, G, I, and K. Full-length blots showing protein expression of APOC3 and DGAT2 under de novo lipogenesis–inducing conditions in vehicle-treated, APOC3-overexpressing (APOC3-OE), and APOC3 knockdown (APOC3-KD) HepG2 cells. Vinculin (VCL) was used as a loading control.
